# The ability to divide spatial attention across non-contiguous locations develops in middle childhood

**DOI:** 10.3758/s13414-025-03182-8

**Published:** 2025-11-25

**Authors:** Tashauna L. Blankenship, Roger Strong, Melissa M. Kibbe

**Affiliations:** 1 Department of Psychology, University of Massachusetts, 100 Morrisey Blvd., Boston, MA 02125 USA; 2 McLean Hospital, Harvard Medical School, Boston, MA USA; 3 Department of Psychological & Brain Sciences, Boston University, Boston, MA USA

**Keywords:** Development, Attention: space-based, Multifocal attention

## Abstract

Adults can effectively divide visual attention across non-contiguous spatial locations. However, it is currently unknown whether the ability to deploy multifocal attention is a hallmark of human endogenous attention, or whether this ability develops with maturation of the neural areas that support deployment of attention across multiple locations. Across two experiments we investigated children’s and adults’ ability to split attention in an adaptation of Awh and Pashler’s (*Journal of Experimental Psychology, 26*[2], 834–846, 2000) task. Participants were cued to attend to two non-contiguous spatial locations in an array of six locations. In Valid trials, participants were probed to report the identity of the digit that appeared briefly in one or both of the cued locations. In Invalid trials, participants were probed to report the identity of the digit that appeared in an uncued location either *between* the two cued locations or *outside* the two cued locations. We reasoned that if participants are able to divide their attention between the two non-contiguous cued locations, they should perform better on Valid compared with Invalid trials, and should perform equally on both types of Invalid trials. We found evidence for multifocal spatial attention in 8-year-olds and adults. However, 6-year-olds appeared to use a strategy consistent with a single focus of attention. Overall, these findings suggest that the ability to divide attention between noncontiguous locations develops during middle childhood.

**Supplementary Information:**

The online version contains supplementary material available at 10.3758/s13414-025-03182-8.

## Introduction

Whether visual attention can be divided across space has been debated (Cavanagh & Alvarez, 2005; Franconeri et al., 2010; Walter, Keitel, Muller, 2016). Classically, visual attention has been argued to be constrained to a single spatial location (Crick, 1984; Posner et al., Posner 1978), giving rise to the single “spotlight” of attention framework (e.g., Kahneman & Treisman, 1984; Yantis, 1992). In this framework, attending to multiple locations requires either spreading attention diffusely to the locations and their intervening space or rapidly switching attention between the two locations. A competing model suggests that rather than being restricted to a single focus, visual attention can have multiple foci (i.e., multifocal attention; Awh & Pashler, 2000; Cavanagh & Alvarez, 2005). Multifocal attention models posit multiple “spotlights” of attention, which can be split between noncontiguous locations while suppressing the intervening space (Awh & Pashler, 2000; Cavanagh & Alvarez, 2005).

Awh and Pashler (2000) directly tested the multifocal attention hypothesis in adults. They showed participants displays with multiple locations in which digits could be briefly presented. On each trial, they cued two of the locations, and then asked participants to identify the digits that were displayed *either* in the cued locations (valid trials) and in other, non-cued locations (invalid trials). Critically, the invalid locations were located *between* the cued locations or *outside* of the cued locations. The logic behind the design was that if visual attention operates using a single focus of attention that is spread between the two cued locations, then the locations in the space between the cued locations should be captured by this single focus, and participants should more accurately report the digits between the cued locations compared with the digits outside of the cued locations. Alternatively, if visual attention operates using multiple foci, attention should be divided, rather than spread, across the cued locations, and participants should show equally poor performance at reporting the digits in any uncued location, regardless of their spatial locations relative to the cued location. They obtained a pattern of results consistent with the multifocal hypothesis: non-contiguous regions of space were attentionally enhanced without enhancing the area between (see also McMains & Somers, 2004; Müller et al., 2003).

Further evidence for the multifocal attention hypothesis comes from multiple object tracking (MOT) studies. Alvarez and Cavanagh (2005) designed a MOT task where participants tracked target objects that were either presented bilaterally (separated between the hemifields of vision) or unilaterally (within one hemifield of vision). Participants could track more moving targets when the targets were presented between visual hemifields compared with within visual hemifields (a “bilateral field advantage”), suggesting independent attentional resources that operate within visual hemifields. Using a similar MOT task, Strong and Alvarez (2020) found that participants performed better when the objects they needed to track moved within the same visual hemifield, and worse when objects crossed over to the opposite hemifield, suggesting that there is an attentional cost incurred when separate attentional mechanisms have to exchange information (a “crossover cost”).

While this previous work has revealed evidence for multiple foci of attention, little is understood about the emergence of the ability to divide attention in human development. On the one hand, the division of attention may be a characteristic hallmark of human visual attention. That is, the ability to divide attention should be observed in any human that can deploy visual attention endogenously. On the other hand, the ability to divide attention may emerge with experience and/or neural maturation. Childhood is a period of rapid neural maturation, including maturation of the corpus collosum (Westerhausen et al., 2011), which is responsible for cross-communication between the left and right hemispheres of the brain (Luck et al., 1989; Qin et al., 2016; Tong, 2004), and has been linked to selective attention in children (Siffredi et al., 2019). Selective attention develops rapidly until around 10 years of age, after which only minor improvements are observed (Klenberg et al., 2001). This developmental plateau may be a product of maturation of the corpus collosum and subsequent improvements in interhemispheric communication. Visual selective attention may require integrating information from the left and right visual fields, which in part involves coordination via the corpus collosum (Hines et al., 2002) and has been argued to play a role in the integration of hemispheric independent visual resources (i.e., multifocal attention; Hines et al., 2002). It is therefore possible that early in development humans may start out with a single focus of attention, and adult-like multifocal attention may be a product of these maturation processes. Examining visual attention during this period of corpus collosum maturation may provide insight into the existence and emergence of independent attentional resources.

There is some evidence to suggest that the ability to track multiple objects via multifocal attention develops between the ages of 6 and 8 years. Blankenship et al. (2020) examined hemifield-related signatures of multifocal attention using a child-friendly adaptation of the MOT task of Strong and Alvarez (2020). They found that the number of objects children could track increased substantially between 6 and 8 years, with 8-year-olds achieving near adult-like tracking capacity. However, evidence of hemifield-independent signatures was mixed, making it less clear how attention was deployed across locations. The extent to which children split attention, and the development of this ability during a crucial period of neural maturation, remains unknown.

In two preregistered experiments, we designed a modified version of Awh and Pashler’s (2000) task to test whether and when children split their attention when attending to two noncontiguous locations. We tested a sample of adults (replicating Awh & Pashler, 2000) and a sample of 6-year-olds and 8-year-olds. We chose to focus on children of these ages since this is a period of substantial maturation of neural areas thought to play a critical role in multifocal attention (e.g., Westerhausen et al., 2011) and previous work showed developmental change in multiple object tracking abilities between the ages of 6 and 8 years (Blankenship et al., 2020). If multifocal attention is a characteristic hallmark of human visual attention, we would expect children to divide attention, with little developmental change in attentional strategies over time. However, if multifocal attention is a biproduct of neural maturation or experience, we might expect developmental differences in attentional strategy use with development.

In Experiment 1, we asked 6- and 8-year-old children and adults to attend to two noncontiguous locations, and then to respond to probes appearing either at those attended locations, between those attended locations, or outside of those attended locations. Following the logic of Awh and Pashler’s (2000) work, if participants are *splitting* attention, they should perform better when probed on one of the two cued locations compared with when they are probed on an uncued location, and they should perform poorly when probed on any uncued location, regardless of whether it is between or outside of the cued locations. However, if participants are *spreading* a focus of attention between the locations, they should perform similarly when probed on a cued location and when probed on an uncued location *between* the two locations, and they should perform better when probed on uncued locations *between* the two cued locations compared with *outside* the two cued locations. Follow-up Experiment 2 was similar in logic to Experiment 1, but was designed to disambiguate between multifocal and potential single-focal strategies in 6-year-olds.

## Experiment 1

### Method

#### Participants

This was a preregistered study (see http://osf.io/65b7u/). Our target sample was 75 participants (25 6-year-olds, 25 8-year-olds, and 25 adults).^1^ The sample size was determined through a power analysis performed by resampling pilot data from a sample of children ranging from 6 to 8 years old (*n* = 13) for each condition (valid, invalid between, invalid outside). This power analysis was based on previous studies (Blankenship et al., 2020; Strong & Alvarez, 2019) who used a similar procedure. Critically, we made an exception to this procedure when the participants scored at 0% for any condition, which mainly occurred in the invalid conditions. Rather than resampling trials when participants had 0% accuracy, we simulated chance performance by doing a weighted coinflip for each trial in that condition, giving each trial a 10% chance level of being answered correctly. This eliminated the possibility of these participants always contributing a score of 0% when resampled. We used this power estimation approach for several reasons. First, it allowed us to estimate the sample size needed in our child samples to simultaneously detect significant differences (using paired-samples *t* tests) in both our comparisons of interest: 1) valid trials versus invalid (between) trials (*d*_z_ = 2.46) and 2) valid trials versus invalid (outside) trials (*d*_z_ = 2.29). Second, it allowed us to account for the unequal number of trials in each of the three experimental conditions. Finally, it allowed us to make the statistically conservative assumption that participants who had 0% accuracy on invalid trials would perform at chance level (10%) in the long run. The results of this power analysis (α =.05) suggested that in order to have .95 power to detect both comparisons of interest within each age group, 25 participants were needed for each age group. Due to overscheduling, we slightly exceeded our target sample, with 79 individuals participating (27 6-year-olds, *M* = 6.42 years, *SD* =.29 years, 13 girls; 26 8-year-olds, *M* = 8.45 years, *SD* =.28 years, 11 girls; and 26 adults, *M* = 29.85 years, *SD* = 8.72, 18 women). An additional five children (four 6-year-olds and one 8-year-old) participated but were excluded from analyses for not completing the task (2), computer error (2), or for completing the task with 100% accuracy, suggesting the possibility that their parent may have completed the task for them (1).

#### Procedure

We adapted Awh and Pashler’s (2000) design to be appropriate for use with children. Participants completed the study in their homes online, and the task was hosted on github (github.com/DevelopingMindsLab/NumberCrunchers). Because participants completed the study from their homes, we were not able to control display size or viewing distance. We include estimates for the dimensions of our stimuli in degrees of visual angle in the Supplementary Materials.

##### Instructions

The task was presented as a computer game. Participants were shown a monster (called a “Number Cruncher”) and were told that their job was to find the Number Cruncher’s favorite numbers. Prior to beginning the task, participants received audiovisual instructions on how to maintain fixation on the cross presented in the center of the screen, how to use their peripheral vision to attend to different locations, and how to make keyboard responses. Following instructions, participants completed eight practice trials (four Valid Single Probe trials, two Valid Double Probe trials, one Invalid Between trial, and one Invalid Outside trial; see Supplemental Material for a detailed description of both the instructions and practice trials). Adults and children completed the same task; we explained to adults that the instructions and task were meant to be child friendly.

##### Task trials

Participants were asked to maintain fixation on the cross during all test trials. On each trial, participants viewed 6 black masks (the digit 8) presented horizontally (three on each side of the fixation cross) for 750 ms. Two of the masks were then cued by turning red (750 ms). The cued masks were always separated by one uncued mask. The masks were then replaced by an array of 6 characters for 250 ms. The characters were then masked again (100 ms). The 250-ms presentation time was determined after piloting with our youngest participants. Children tend to orient slower than adults (e.g., Yang et al., 2002 note that 6 year-olds took ~ 400 ms to orient to stimuli), so a slower speed would likely be sufficient for our younger participants. Fixation to the center of the screen is critical to promote multifocal attention (rather than rapidly switching between locations). Then, either one (Valid Single Probe) or both (Valid Double Probe trials) of the cued masks was probed, or an uncued mask either between the cued masks or outside one of the cued masks was probed (Invalid Probe trials) (relevant mask(s) turned red to prompt response). Participants were asked to report the digit that had appeared at the probed location(s) by entering the digit using their keyboard,^2^ after which an animated Number Cruncher appeared with either a smile or neutral face depending on the participants’ responses (smile = correct; neutral = incorrect).

**Fig. 1:**
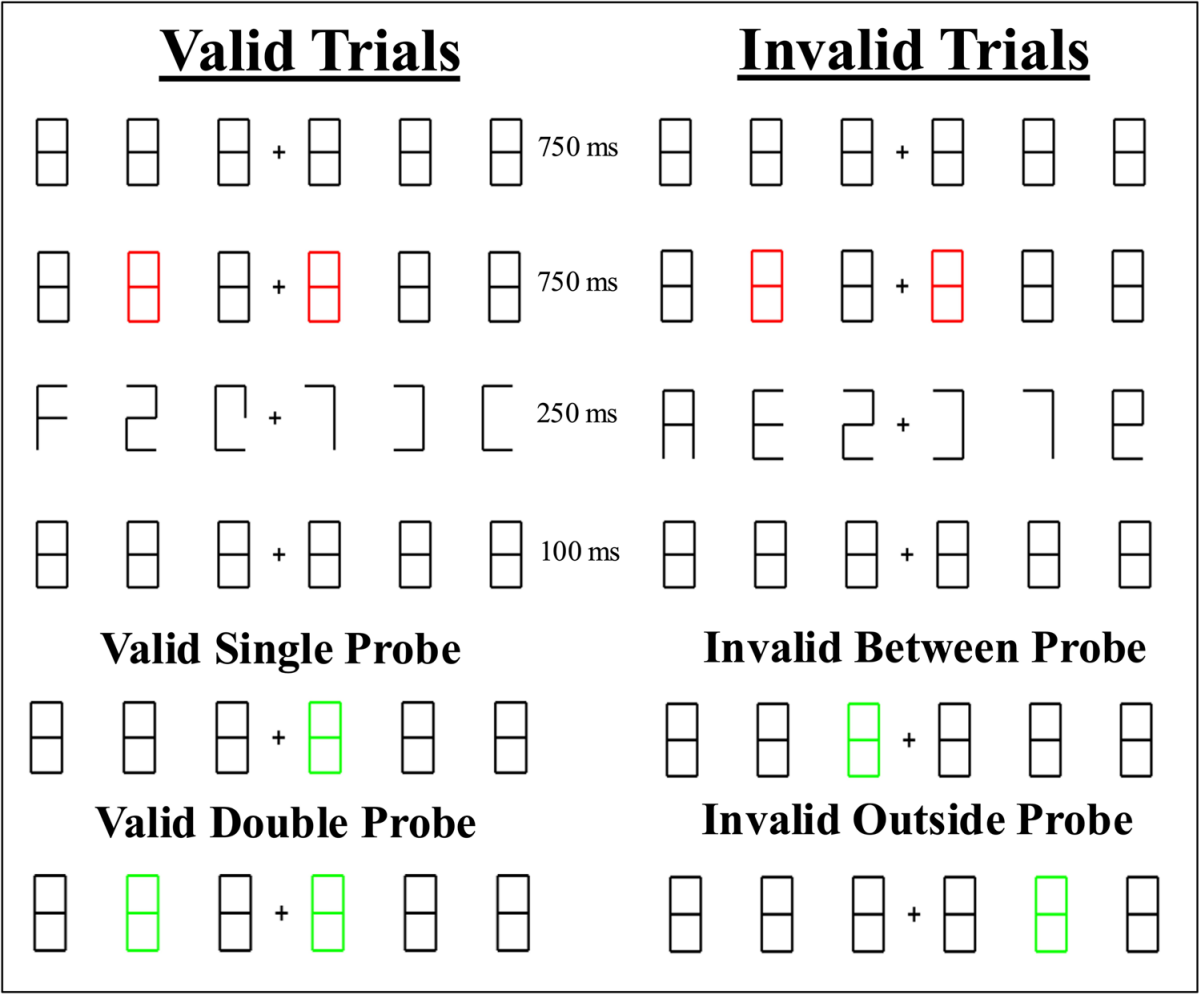
Example stimuli and time course for Valid (Single and Double Probe) and Invalid (Between and Outside) trials in Experiments 1 and 2. *Note.* Double Probe trials were only used in Experiment 1. (Color figure online)

Participants completed a total of 30 trials (two blocks of 15 each, with a different Number Cruncher character for each block; Fig. 1): 16 Valid Single Probe trials (in which one of the cued locations was probed), 10 Valid Double Probe trials (in which both cued locations were probed), 2 Invalid Between trials (in which the location between the two cued locations was probed), and 2 Invalid Outside trials (in which a location outside of one of the cued locations was probed). Invalid trials made up a small proportion (13.3%) of the total number of trials to ensure that children were attending only to the cued locations. Keeping the proportion of invalid trials low reduced the possibility that children would shift their strategy to attend to more locations. A similar approach was taken by Awh and Pashler (2000), whose invalid trials also made up a small proportion of their total trials (20%). However, because their participants were adults, they were able to run more trials. We included a smaller number of total trials in order to reduce the possibility of participant fatigue, which also meant that we used only a small total number of invalid trials.

Valid and Invalid trials appeared randomly within each block. Valid Double Probe trials were included to verify that participants were attending to both locations, but these trials were not included in analyses comparing valid and invalid trials. In Valid Double Probe trials, participants were asked to respond to the leftmost probe first, then to the rightmost probe. A question mark appeared beneath the left and then right probe, to help ensure that participants responded in the correct order.

Participants received a score of 1 (correct) or 0 (incorrect) for each trial. For the Double Valid Probe trials, participants were only given a score of 1 if they got *both* probe locations correct; if they were correct on one and not another then they received a score of 0. We used accuracy on both probe locations as a conservative estimate, relative to the Valid Single Probe trials, of multifocal ability; if participants are engaging in multifocal strategies then they should get both locations correct at rates similar to the Valid Single Probe trials.

### Results

Data for Experiments 1 and 2 are available online (http://osf.io/65b7u/).

#### Do children spread or split attention?

Following our preregistered analysis plan, we first asked whether participants performed differently on Valid Single Probe compared with the Invalid Probe trial types as a function of age. We conducted a repeated-measures analysis of variance (ANOVA) on participants’ mean proportion correct with Trial Type (Valid Single Probe, Invalid Between Probe, Invalid Outside Probe) as a within-subjects factor and Age Group (6-year-olds, 8-year-olds, or adults) as a between-subjects factor. We observed a significant main effect of Trial Type, *F*(2, 152) = 133.79, *p* <.001, η_p_^2^ =.638, a significant main effect of Age Group, *F*(2, 76) = 17.07, *p* <.001, η_p_^2^ =.310, and a significant Trial Type × Age Group interaction, *F*(4, 152) = 5.97, *p* <.001, η_p_^2^ =.136. Planned paired *t* tests comparing performance on Valid Single Probe trials to both Invalid trial types at each age group revealed that all age groups performed significantly better on Valid compared with Invalid trials (all *p* <.001). While not pre-registered, we also conducted Bayesian ANOVAs (JASP, 2024; default priors) to accompany the frequentist ANOVAs because they can provide useful insights into the strength of our results. A Bayesian ANOVA revealed that the model including the Trial Type × Age Group interaction (more than 1 billion times more likely than the null), was about 1000 times more likely than the model including only the main effects of Trial Type and Age Group.

**Fig. 2:**
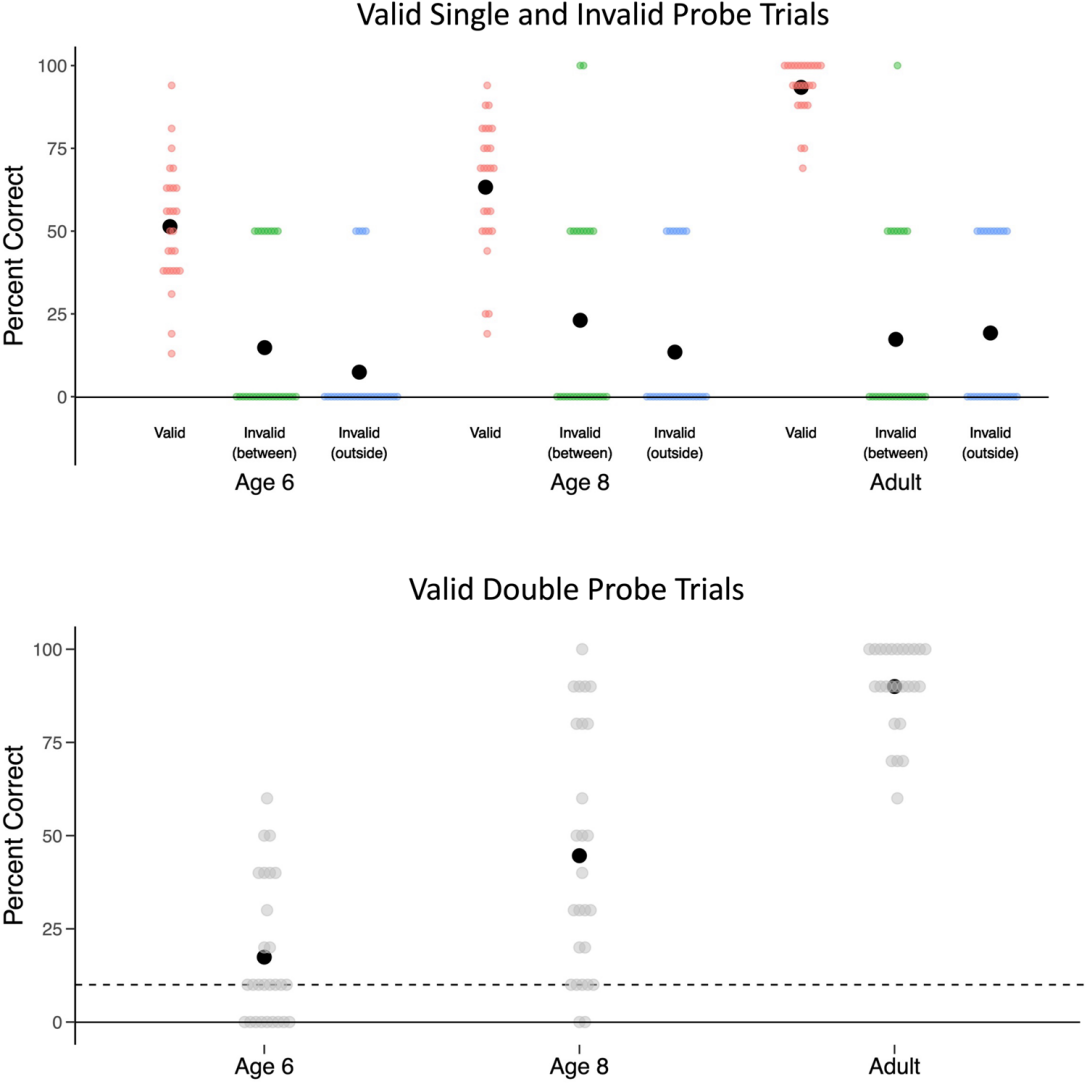
Top panel shows percentage correct performance on Valid Single Probe (red), Invalid Between (green), and Invalid Outside (blue) Single Probe trials as a function of age. Bottom panel shows the percentage of Double Valid Probe trials in which participants responded correctly for both probes. The width of the individual score lines corresponds to the distribution of scores (wider lines indicate more participants received that score). The black circles represent mean performance for each age on each trial type. The dashed line in the bottom panel represents chance-level performance (10%). (Color figure online)

We followed up the Age × Trial Type interaction with separate one-way ANOVAs on mean Valid Single Probe trial performance and mean Invalid Single Probe trial performance with Age Group (6-year-olds, 8-year-olds, and adults) as a between-subjects factor. We found that performance in Valid Single Probe trials increased with age, *F*(2, 76) 45.41, *p* <.001, η_p_^2^ =.544, and a Bayesian ANOVA revealed that the model including the main effect of Age was over 1 billion times more likely than the null model. By contrast, performance in the Invalid trials was similar across age groups, *F*(2, 76) = 1.50, *p* =.23, η_p_^2^ =.038, and a Bayesian ANOVA (not preregistered) revealed the null model was 3 times more likely than the model including Age (see Fig. 2).

We then asked whether participants performed differently in the two Invalid Single Probe trial types using a repeated-measures ANOVA with Invalid Trial Type (Between or Outside) as a within-subjects factor and Age Group (6-year-olds, 8-year-olds, or adults) as a between-subjects factor. We observed no main effect of Invalid Trial Type, *F*(1, 76) = 1.51, *p* =.224, η_p_^2^ =.019, no main effect of Age Group, *F*(2, 76) = 1.50, *p* =.230, η_p_^2^ =.038, and no Invalid Trial Type × Age Group interaction, *F*(2, 76) =.736, *p* =.482, η_p_^2^ =.019. A Bayesian ANOVA (not preregistered) revealed that the null model was about 3 times more likely than the data under the model including a main effect of Trial Type, providing anecdotal evidence in favor of the null (Jeffreys, 1961). These results, visualized in Fig. 2, suggest participants, regardless of age, may have split their attention between the cued locations rather than spread attention across the cued locations.

To confirm that our results were not driven by differences in number of trials between invalid (four trials) and valid (16 trials) trials, we ran another repeated-measures ANOVA on mean percentage correct with Trial Type (Valid Single Probe, Invalid Outside, Invalid Between) as a within-subjects factor, but only using participants’ performance on the first two Valid Single Probe trials. Results were consistent with our previous analyses. We observed a significant main effect of Trial Type, *F*(2, 152) = 89.952, *p* <.001, η_p_^2^ =.542, a significant main effect of Age Group. *F*(2, 76) = 10.876, *p* <.001, η_p_^2^ =.223, and a significant Trial Type × Age Group interaction, *F*(4, 152) = 4.316, *p* <.01, η_p_^2^ =.102. A Bayesian ANOVA revealed that the model including the Trial Type X Age Group interaction (more than 1 billion times more likely than the null), was about 40 times more likely than the model including only the main effects of Trial Type and Age Group. Paired *t* tests comparing performance on Valid Single Probe trials to both Invalid trial types at each age group revealed that all age groups performed significantly better on Valid compared with both types of Invalid trials (all *p* ≤.001).

#### Comparing performance to chance

As preregistered, to confirm that participants attended to the cued locations, we compared participants’ mean percentage correct in both the Valid Single Probe and Valid Double Probe trials to chance levels (Valid Single: 0–9 possible responses; chance = 10%; Valid Double: 0–9 possible responses for each of the two locations; chance of getting both correct = 10% × 10% = 1%^3^) using two-tailed one-sample *t* tests and Bayes factor analysis. Participants, regardless of age, responded correctly at rates significantly above chance levels in both Valid Single Probe and Valid Double Probe trials, suggesting that they did indeed attend to both locations on some trials. These results are summarized in Table 1.

**Table 1:** Results of two-tailed one-sample *t* tests comparing 6- and 8-year-old children’s and adults percentage correct to chance for Valid Single Probe (chance = 10%) and Valid Double Probe (chance = 1%) trials

	6-year-olds			8-year-olds			adults		
Condition	*M* % correct	*t*	*JZS BF*_*10*_	*M* % correct	*t*	*JZS BF*_*10*_	*M* % correct	*t*	*JZS BF*_*10*_
Valid Single	51.37%	11.77	> 1 billion	63.27	13.50	> 1 billion	93.42	48.61	> 1 billion
Valid Double	17.41%	4.50	200	44.62	6.71	38,461.54	90.00	38.91	> 1 billion

All *p*s <.001. Jeffreys–Zellner–Siow (JZS) Bayes Factors (JASP, 2024; Cauchy scale set to 1) show the odds of the alternative over the null

#### Distinguishing single-focal and multifocal attention strategies

Our preregistered chance levels presume that participants failed to attend to the probed location(s), and therefore, they guessed when prompted to respond. Our results suggest that participants overall were not simply guessing. However, above-chance performance does not allow us to distinguish between multifocal strategies, in which participants attend to *both* locations, and single-focal strategies, in which participants attend to *one* location only. We conducted an exploratory analysis to investigate whether participants’ performance could be distinguished from a single-focal strategy. We reasoned that, if participants relied on a single-focal strategy, then we can assume they will respond correctly when probed on the attended location 100% of the time, and guess when probed on the other, unattended location (resulting in at best 10% accuracy; see Blankenship et al., 2020; Trick et al., 2005, for similar logic). While we recognize that 100% accuracy is unlikely, especially in children, we used perfect accuracy on one cue to provide a conservative estimate of single focal performance.

On Single Valid Probe trials, two locations are cued, and each location (rightmost, leftmost) is probed on half of the trials. Therefore, we can use the following formula for expected performance if participants are using a single-focal strategy in Single Valid Probe trials: (100% × 50%) + (10% × 50%) = 55%. We then compared participants’ performance on Single Valid Probe trials to 55% using one-sample *t* tests for each age group (see Table 2). We found that 8-year-olds’ and adults’, but not 6-year-olds’, performance on Single Valid Probe trials was significantly higher than would be expected from a single-focal attention strategy, while 6-year-olds’ performance could not be distinguished from what would be expected if they were using a single-focal strategy.

**Table 2:** Results of two-tailed one-sample t-tests comparing participants’ percentage correct to chance calculated for single-focal strategies for Valid Single Probe (chance = 55%) and Valid Double Probe (chance = 10%) trials

	6-year-olds			8-year-olds			adults		
Condition	*M* %	*t*	*JZS BF*_*10*_	*M* %	*t*	*JZS BF*_*10*_	*M* %	*t*	*JZS BF*_*10*_
Valid Single	51.37	−1.03	.25	63.27	2.09	1.08	93.42	22.39	1.28e + 15
Valid Double	17.41	2.03	.96	44.62	5.33	1459.85	90.00	34.98	4.08e + 19

JZS Bayes Factors (JASP, 2024; Cauchy scale set to 1) show the odds of the alternative over the null

On Double Valid Probe trials, two locations are cued, and both locations are probed. A single-focal strategy in Double Valid Probe trials would result in (100% × 10%) = 10% accuracy. We compared participants’ performance in Double Valid Probe trials to 10% (see Table 2; Fig. 2). Again, we found that 8-year-olds’ and adults’ performance was consistent with attending to both locations, while 6-year-old’s performance was not distinguishable from what would be expected from a single-focal strategy. Critically, accuracy on Double Valid Probe trials was defined as correct responses on *both* probed locations. Exploratory analyses suggested that participants at each age group performed better on left than right probed locations (paired-samples *t* tests; *p*s <.05), but that performance was above chance (.1) for both locations (one-sample *t* tests; *p*s <.05). This result is consistent with previous work suggesting a left-side advantage (Holcombe et al., 2017; Ransley et al., 2018), and suggests that although 6-year-olds performance was consistent with single focal strategies, they were able to maintain both left and right location identities at levels above what would be expected by chance.

### Discussion

In Experiment 1, we examined whether children could reliably attend to two non-contiguous locations using multifocal attention. In order to address this question, we designed a child-friendly version of an adult task used to examine multifocal attention (Awh & Pashler, 2000). We examined multiple signatures of multifocal attention: 1) better performance at cued locations than at areas in between cued locations, 2) similar performance at areas in between cued locations and areas outside of cued locations, and 3) better performance at cued locations than would be expected from only attending to one cued location. Our results suggest that 8-year-olds and adults could reliably attend to two locations. Further, while the strategies used were less clear (i.e., small BF and numerical trend of larger between than within performance) the lack of difference may suggest use of splitting rather than spreading strategies. Together these results suggest some developmental continuity in the deployment of multifocal attention between childhood and adulthood.

However, our results for 6-year-olds were less clear. Like 8-year-olds and adults, 6-year-olds were above chance on Valid Single Probe trials and performed similarly whether the probed location was between or outside the cued locations, consistent with what would be expected if children were splitting attention between the two cued locations. However, it was unclear whether 6-year-olds were truly using multifocal attention in the task, or whether they were attending to only one of the two cued locations on each trial. Our formula for expected performance under a single- or multifocal strategy assumes perfect performance for the attended location(s) and guessing for the unattended location(s). This approach may have underestimated children’s performance. Six-year-olds may have been using multifocal attention but may not have achieved perfect performance for the attended location(s), which resulted in overall performance that was not distinguishable from a single-focal strategy.

In Experiment 2, we sought to examine whether 6-year-olds are indeed using multifocal attention in the task. To do so, we obtained a baseline measure of each child’s ability to respond with the correct digit when probed: children completed a series of trials in which only a single location was cued and subsequently probe, in addition to trials in which two locations were cued (as in Experiment 1). We then used their individual performance on these Single Cue trials to compute individualized single-focal chance levels (for a similar approach in a multiple object tracking task, see Blankenship et al., 2020).

## Experiment 2

### Methods

#### Participants

This study was preregistered. The preregistration documents can be found online (http://osf.io/65b7u/). Our target sample was 25 6-year-olds determined through the power analysis outlined in Experiment 1 (Strong & Alvarez, 2019). Data were collected online and the task was hosted over github (github.com/DevelopingMindsLab/NumberCrunchers2). Our final sample included 25 6*-*year-olds (*M* = 6.37 years, *SD* =.30 years, 10 girls).

#### Procedure

The procedure used was similar to Experiment 1, in which children completed Valid Single Probe and Invalid Single Probe trials, except that we did not include Valid Double Probe trials. Instead, children completed trials in which we cued and probed only one location (Single Valid Cue trials) in order to get a baseline measure of children’s ability to respond with the correct number when attending to only one location.

##### Instructions

The instructions were similar to Experiment 1, except that instead of receiving instructions on how to respond to Valid Double Probe trials, participants received instructions on how to attend and respond to Single Valid Cue trials (see Supplemental Material for a detailed description of both the instructions and practice trials).

##### Task trials

**Fig. 3:**
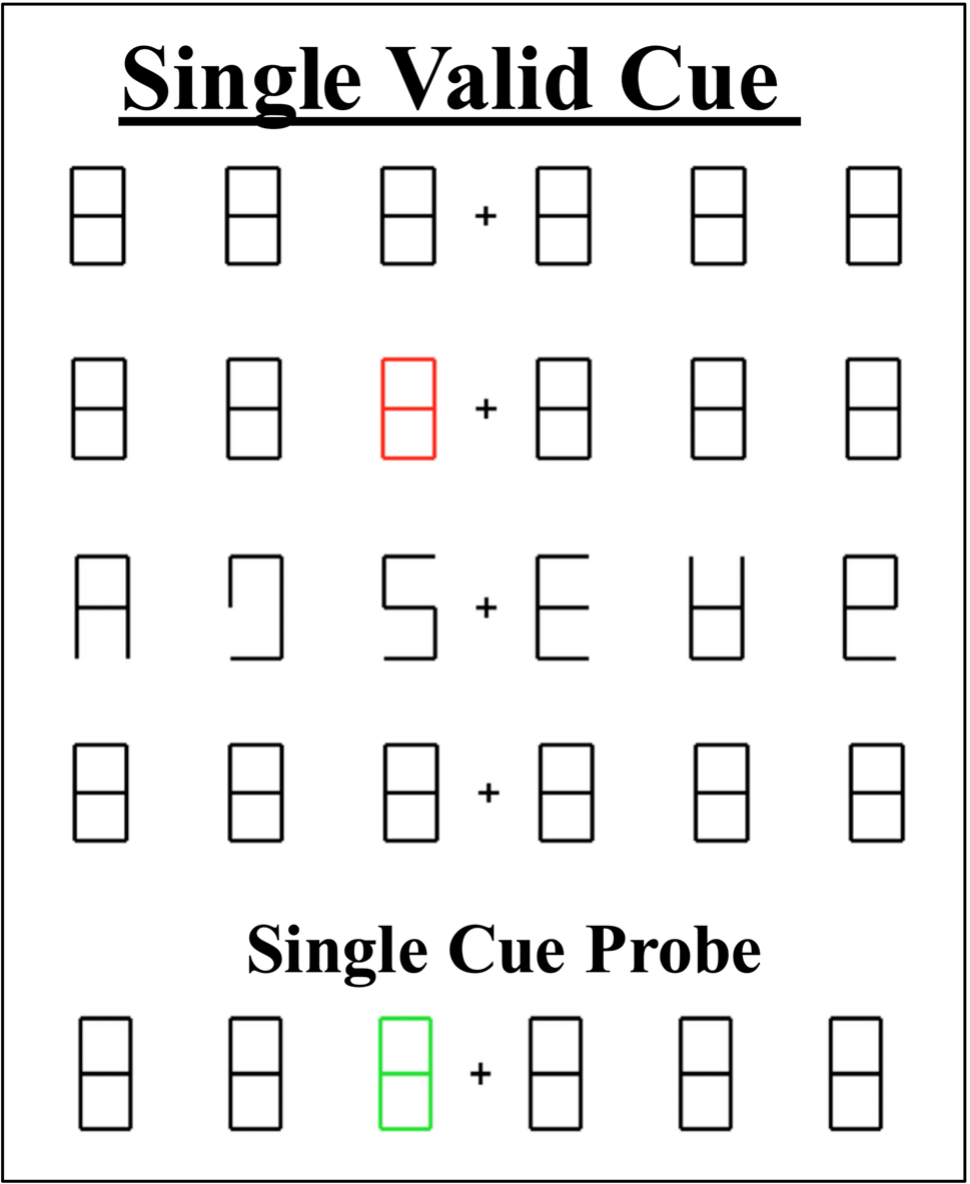
Example stimuli and time course for Single Valid Cue trials in Experiment 2. (Color figure online)

The time course of each trial was the same as in Experiment 1. Children completed 30 test trials. Sixteen trials (53.3%) were Valid Single Probe trials, in which two locations were cued and one of those locations was probed. Four trials (13.3%) were Invalid Single Probe trials, in which two locations were cued and an uncued location (either between the two cued locations (two trials) or outside of the cued locations (two trials) was probed. Ten trials (33.3%) were Single Valid Cue trials, in which only one location was cued and then subsequently probed (see Fig. 3 for Single Valid Cue trial example and Fig. 1 for Valid Single Probe and Invalid trials examples). These trials were used to generate a baseline for single focal performance. Trials were presented in two blocks of 15 trials each, with a different Number Cruncher characters for each block. The three trial types (Valid Single Probe, Invalid Single Probe, and Single Valid Cue) trials appeared pseudorandomly within each block.

### Results

#### Do 6-year-olds spread or split attention?

**Fig. 4:**
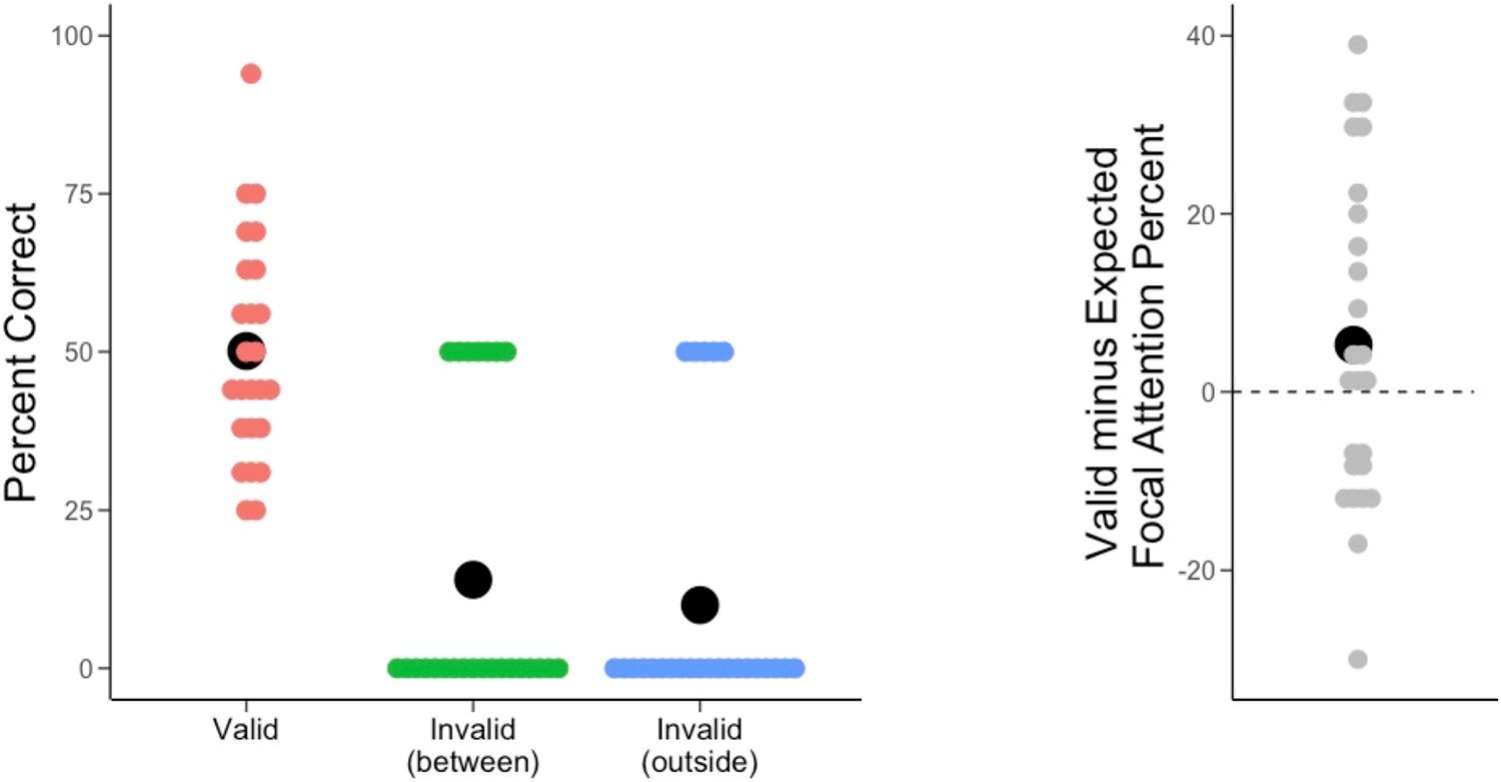
Six-year-olds’ percentage correct (left figure) performance on Valid Single Probe and Invalid trial types. The right figure shows the difference between children’s actual Valid Single Probe performance and children’s expected performance if they were only attending to one location (i.e., using a single focal strategy). Note the mean value is not significantly different from 0. The black circles represent means. The width of the score lines represent score distribution (i.e., longer line means more participants scored that value). (Color figure online)

Following our preregistered analysis plan, we ran a repeated-measures ANOVA on mean percentage correct with Trial Type (Valid Single Probe, Invalid Outside, Invalid Between) as a within-subjects factor. This revealed a main effect of Trial Type, *F*(2, 48) = 25.576, *p* <.001, η_p_^2^ =.516, and a Bayesian ANOVA revealed that the model including Trial Type was more than 1 billion times more likely than the null model. Follow-up Bonferroni-corrected paired-samples *t* tests revealed that 6-year-olds performed significantly better on Valid Single Probe trials compared with both Invalid trial types (*p*s <.001), and children’s performance in Invalid Between and Invalid Outside trials were not statistically different, *t*(24) =.57, *p* =.574; JZS BF_10_ =.18 (see Fig. 4).

As in Experiment 1, we also conducted a repeated-measures ANOVA on mean percentage correct with Trial Type (Valid Single Probe, Invalid Outside, Invalid Between) as a within-subjects factor only using performance on the first two Valid Single Probe trials. The results were consistent with the analysis of the full set of trials. We observed a main effect of Trial Type, *F*(2, 48) = 14.908, *p* <.001, η_p_^2^ =.383. A Bayesian ANOVA revealed that the model including Trial Type was more than 20,000 times more likely than the null model. Follow-up Bonferroni-corrected paired-samples *t* tests revealed that 6-year-olds performed significantly better on Valid Single Probe trials compared with both Invalid trial types (*p*s ≤.001).

#### Comparing performance to chance

As preregistered, we compared mean percentage correct for the Valid Single Probe (two locations cued and one probed) and Single Valid Cue (one location cued and one probed) trials to chance levels (10%; 0–9 possible responses) using both two-tailed one-sample *t* tests and Bayes factor analysis (JASP, 2024; Cauchy scale set to 1). Six -year-olds were significantly above chance levels for both Valid Single Probe (*M* = 50.12%), *t*(24) = 11.50, *p* <.001, JZS BF_10_ > 1 billion, and Single Valid Cue trials (*M* = 79.68%), *t*(24) = 16.96, *p* <.001, JZS BF_10_ > 1 billion.

#### Distinguishing single-focal and multifocal attention strategies

As preregistered, we computed a single focal performance level titrated to each child’s performance in the Single Valid Cue trials, which allowed us to account for individual differences in single focal ability. For example, if a child performed at 70% in Single Valid Cue trials, and they attended to only one location during Valid Single Probe trials, that participant is expected to achieve 70% accuracy on the trials in which they attended to the location that was probed, and 10% on trials where the other location was probed. For this participant, their anticipated performance if they only attended to one location would be calculated by (70% ×.50) + (10% ×.50) = 40%. We used a paired samples t-test to compare these titrated single-focal chance values to children’s Single Valid Probe trial performance. We found that 6-year-olds’ accuracy on Single Valid Probe trials was not significantly different from their expected performance if they used a single focal strategy, *t*(24) = 1.43, *p* =.165, JZS BF_10_ =.40. These results are depicted in Fig. 4, right panel.

### Discussion

In Experiment 2, we examined 6-year-olds’ ability to attend to multiple non-contiguous locations and their use of single focal strategies. Six-year-olds were above chance levels on both Valid Single Probe trials, in which two locations were cued and one was probed, and Single Valid Cue trials, in which one location was cued and subsequently probed, suggesting that they could attend to at least one location reliably. However, we were primarily interested in 6-year-olds’ ability to deploy multifocal strategies to attend to multiple locations. To this end, we computed an estimate of each participant’s expected performance on Valid Single Probe trials if they were only attending to one location based on their performance when they were asked to attend to only one location. This allowed us to account for children’s individual differences in errors when attending to a single location. We found that 6-year-olds’ performance when tasked with attending to two non-contiguous locations was not significantly different than would be expected if they were attending to only one of the two locations. Our results also suggest that children were attending to the cued locations, since they performed significantly better on valid compared with invalid trials. However, the exact strategies used are less clear. It is possible that 6-year-olds are using a single focal strategy, potentially shifting a single spotlight of attention between two cued locations.

Interestingly, inspection of Fig. 4’s right panel shows there was significant variability in 6-year-olds’ performance, with some 6-year-olds showing performance on valid single probe trials above what would be expected if they were using a single focal strategy. To explore this, we split the sample into high and low performers using a median split based on the difference score comparing valid single probe trials and expected single focal strategy performance (median =.015). We then ran an ANOVA with invalid conditions as a within subject factor and performance on valid single probe trials (high performers vs. low performers) as a between-subject factor. Results suggested that there was a significant interaction between invalid conditions and performance, *F*(1, 23) = 5.982, *p* <.05, η_p_^2^ =.135, no other effects were significant; children who performed above the median on valid trials (based on comparison to expected single cue performance) tended to have higher means on the invalid between trials (*M* =.214) than those who scored below the median (*M* =.036). While exploratory, this may suggest that children who were more likely to attend to both cued locations were also more likely to spread their attention, but further research is needed to better understand the visual attention strategies used in 6-year-olds with consideration of possible individual differences in strategy use. Implications of our results are outlined in the general discussion.

## General discussion

We examined the development of multifocal spatial attention in 6-year-olds, 8-year-olds, and adults using a child-friendly version of the task of Awh and Pashler (2000). Participants were asked to attend to two non-contiguous locations and report the identity of a digit that appeared either in one or both of the two cued locations (valid trials) or between or outside of the cued locations (invalid trials). Following the logic of Awh and Pashler (2000), we reasoned that, if participants attended the two cued locations, they should be able to successfully identify the digits at the cued locations at rates significantly higher than at the uncued locations. Further, if participants are *splitting* their attention between the two locations, rather than spreading attention across the two locations, including the intervening space, they should perform similarly when probed on an uncued location that fell between the two locations and when probed on an uncued location that fell outside the cued locations.

We found that 8-year-olds and adults both showed signatures of multifocal attention: Their performance suggested they split attention between the two locations, suggesting multiple foci of attention deployed to the cued locations. These results suggest adult-like multifocal attention is present by at least age 8. We did not, however, find evidence of multifocal attention in our youngest age group. Instead, 6-year-olds’ performance on the task was consistent with single-focal strategies. These results support that the ability to effectively engage in multifocal visual attention increases across childhood and may reflect continued maturation of neural networks that are essential to visual attention (e.g., Betts et al., 2006; Qin et al., 2016; Tong, 2004).

While we did not observe reliable division of attention in 6-year-olds in our task, these results do not necessarily show that 6-year-olds are *incapable* of multifocal attention. A previous study (Blankenship et al., 2020) found that 6-year-olds were able to use multifocal attention to successfully complete a multiple object tracking task that could not be accomplished using single-focal strategies. However, that study also found substantial developmental increases between 6 and 8 years in multiple object tracking abilities, with younger children able to track fewer objects and to do so less reliably than older children and adults. Taken together with the results of the current experiments, it is possible that the ability to divide attention between non-contiguous locations may be emerging in 6-year-olds. Six-year-olds in our task may have divided attention on some trials and deployed a single focus of attention on other trials, or children may have attempted to divide attention but struggled to do so and consequently were reduced to a single focus of attention or potentially spread attention in some cases (as suggested by an exploratory analysis), consistent with the results of Blankenship et al. (2020). Another possibility is that the structure of the task itself may have masked children’s abilities: the short exposure time (250 ms) may have been too challenging for 6-year-olds to process both digits, and the inclusion of only a few invalid trials may have resulted in noisier data from these younger children. Further, while practice trials were given to ensure children understood the task prior to starting, 6-year-olds might have struggled recognizing digits or following the instructions. More work is needed to better understand moment-to-moment deployment of attention in tasks that require division of attention in younger children.

We intentionally reduced the number of invalid trials to limit children from using alternate attentional strategies (i.e., attending to the uncued locations), but this design decision might have limited our ability to detect differences between the invalid conditions. To account for this possibility, we reran our analyses including only the first two valid single probe trials (Experiments 1 & 2). We found a similar pattern of results as when all of the Valid trials were included, suggesting that two Valid trials were enough to at least detect a difference between valid and invalid trials. Regardless, future work should attempt to include a larger number of trials, both valid and invalid. Due to the COVID-19 pandemic, we also had to move our task online rather than in person. This impacted our ability to monitor participants and ensure compliance (e.g., maintaining fixation). We provided all participants with video instructions which included audio and visual explanations, but it is possible children did not comply. Their high accuracy on valid compared with invalid trials would suggest they maintained fixation, but again we cannot be certain. Additional work should incorporate eye tracking to ensure fixation during task administration.

Our adaptation of Awh and Pashler’s (2000) task required children to attend to cued locations and to rapidly identify the digits at the cued locations. The need to rapidly (250 ms) acquire and then maintain in working memory the *identities* of the digits across two locations may also have been particularly difficult for 6-year-olds. While 6-year-olds performed well on the task when they were tasked only with attending to and reporting one of the digits, they were not perfect. The challenges of attending and identifying may further tax these children’s more limited attentional resources. While our results suggested that 6-year-olds were able to maintain the identity of both probed digits, further work is needed to investigate sources of attentional demands and how those demands might impact the allocation of spatial attentional resources to non-contiguous locations across development. Finally, while we can generate rough estimates of viewing distance (see Supplemental Material), we do not know the exact distance each participant was from their computer screens during task administration. Future studies should include measures of viewing distance (e.g., virtual chinrest; Li et al., 2020) to help control for individual differences in visual angle.

## Summary

We found evidence for multifocal spatial attention in 8-year-olds and adults. When 8-year-olds and adults are attending to two locations they split their attention across the two locations rather than spreading a single focus of attention between the locations. Our results further suggest that 6-year-olds may rely on single rather than more mature multifocal strategies when attending to two spatial locations. Together, these results suggest that the ability to divide spatial attention to multiple, non-contiguous locations may be emerging around age 6, and undergoes significant development between 6 and 8 years of age.

## Supplementary Information

Below is the link to the electronic supplementary material.

Supplementary file1 (DOCX 52 kb)

## Data Availability

The data and materials for all experiments are available and both Experiments 1 and 2 were preregistered: http://osf.io/65b7u/

## Footnotes

1 The preregistration documents are separated based on age. Originally, we proposed 50 participants (25 6-year-olds and 25 adults). The sample of 8-year-olds was added after the onset of initial data collection, so the documents preregistering the age groups are separate.

2 Because the digit 8 was used as a mask, we did not use the digit 8 as a possible digit in a probed location. However, participants were never explicitly told that 8 was not a possible response. Therefore, their responses could consist of any digit between 0 and 9, inclusive.

3 Note that there is a typographical error in our preregistration document, in which the chance level for Valid Double Probe trials is erroneously given as 10% × 10% = 5% instead of 1%.

## Code availability

Task code is available for all experiments online at: github.com/DevelopingMindsLab/NumberCrunchers.
