# Supplementary material for: The ability to divide spatial attention across non-contiguous locations develops in middle childhood
Source: Atten Percept Psychophys. 2025 Nov 25;88(1):9. doi: 10.3758/s13414-025-03182-8 (PMC12647209; doi:10.3758/s13414-025-03182-8)
Supplement: Supplementary file 1 — Supplementary file1 (DOCX 52 kb) [file 13414_2025_3182_MOESM1_ESM.docx]

**The ability to divide spatial attention across non-contiguous locations develops in middle childhood**

**Supplemental Material**

**Stimulus Measurements**

Because all participants completed the experiment remotely via the web, we were not able measure monitor size or participant viewing distance; thus all stimulus measurements are reported in display points (rather than degrees of visual angle. One each trial, children viewed 6 black 8s (masks; height = 104 pixels, width = 54 pixels) which were displayed to the right and left of a “plus sign” (fixation height = 20 pixels, width = 20 pixels) at the center of the screen. The distance between the midpoints of adjacent masks was 150 pixels (see Figure S1).


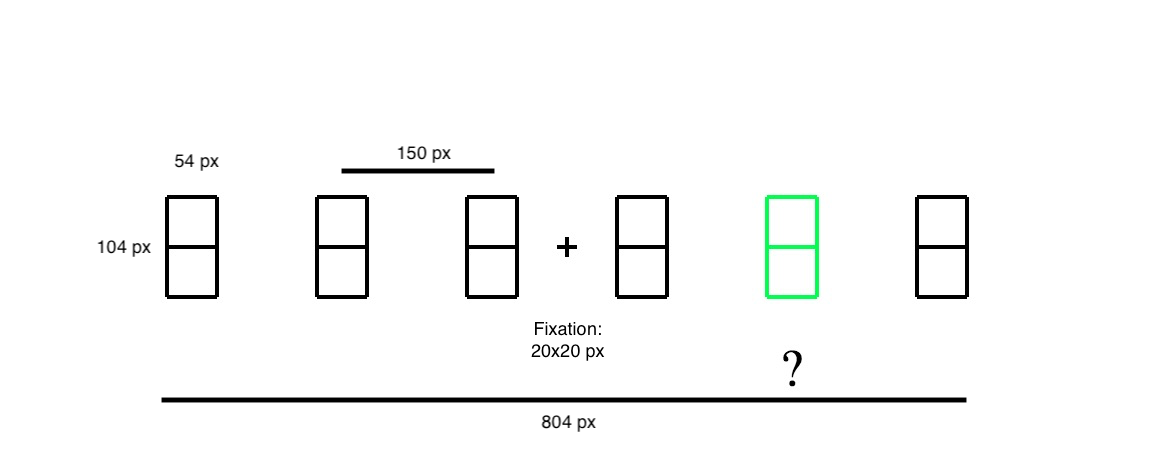


Figure S1: Display measurements for stimuli presented during response period.

To give a rough estimate of measurements in degrees of visual angle, a participant viewing a display with height 8.8 inches, width 13.6 inches, resolution 127 pixels/inch, and viewing distance 21 inches would observe 48.1 pixels per degree of visual angle. Therefore, dividing our measurements reported in display points by 48.1 provides a rough estimation of degrees of visual angle (16.72). This would result in an estimated 3.12 degrees of visual angle between each stimulus for an adult participant. If instead, the participant had a viewing distance of 18 inches (estimated viewing distance for a child), and the same display and resolution, we would observe a viewing distance of 41.7 pixels per degree of visual angle. Using this estimate the degrees of visual angle for the entire display would be 19.28 degrees and 3.60 degrees for the distance between two stimuli. A demonstration of the experiment, including the underlying JavaScript code, can be viewed at: <https://developingmindslab.github.io/NumberCrunchers/NumberCruncherDemo.html>.

**Experiment 1 Instruction Details**

Participants were instructed that the goal of the game was to find the “Number Crunchers” (cartoon monsters) favorite numbers. Six black 8s (masks) were then displayed to the right and left of a “plus sign” (fixation) at the center of the screen. Critically, participants were told to keep their eyes on the plus sign and to use their “side vision” to pay attention to the 8s to the left and right of the plus sign. Two of the 8s would then turn red (cues). Participants were told to pay extra close attention to the red 8s because the Number Cruncher’s favorite number(s) would usually be hiding behind one of those 8s. The masks were then replaced with an array of 6 characters, two of which were numbers. Participants were shown the numbers and the audio instructions emphasized the numbers in the location where the red 8s were previously. The black 8s then replaced the array and one of the masks that was originally cued turned green (probe) and a question mark appeared below it. Participants were asked if they remembered the number “hiding” behind the green 8 (single probe). After a brief (~1 second) pause, the correct answer was revealed, and participants were told that they could use their keyboard to respond during the game. Participants then moved on to the next trial demonstration.

This demonstration started very similarly to the first. Participants were reminded to keep their eyes on fixation and to use their peripheral vision to pay attention to the masks on either side of the fixation cross. Again, participants were instructed to pay extra close attention to the red 8s, and the masks were replaced with 6 characters. Black 8s replaced the array both 8s that were previously cued turned green and a question mark appeared below them. Participants were asked to remember what both numbers were and instructed that they would need to respond starting with the probe furthest left on the screen and moving to the probe on the right. During the left to right instructions, participants observed an arrow pointing to the left probe first and then the right, to emphasize the order of response. They then observed the correct numbers, with the left appearing slightly before the right probe. Following demonstrations, participants completed practice trials at the same speed of the test trials. Prior to practice, participants were instructed that the game would move much faster than the demonstrations “Now let’s practice the game. During the actual game, everything is going to go much faster so pay extra close attention.” Participants completed 8 practice trials (4 valid single probe, 2 valid double probe, 1 invalid between and 1 invalid outside), Following each response, they received visual feedback on if their responses were correct (Number Cruncher smiled) or incorrect (Number Cruncher surprised). Participants were never explicitly told that invalid trials (probed mask not in location of cue) were going to occur. This was intentionally done to limit strategies that might break attention to the cued locations.

**Experiment 2 Instruction Details**

The instructions and practice for Experiment 2 was identical to Experiment 1, except for the double probed trials. Instead of double probed instructions and practice, participants were given instructions on single cue and subsequent single probe trials. Following the double cue and single probe instructions, participants were again reminded to keep their eyes on fixation and to use their peripheral vision to pay attention to the masks on either side of the fixation cross. Participants were then instructed to pay extra close attention to the single red 8 (single cue). The masks were replaced with 6 characters and then the location of the original cue turned green, and participants were asked if they remembered the number “hiding” behind the green 8 (single probe). After a brief (~1 second) pause, the correct answer was revealed, and participants were reminded that they could use their keyboard to respond during the game. Practice was again very similar to Experiment 1, except valid double probe trials were replaced with single cue probe trials. Participants completed 8 practice trials (4 valid single probe, 2 single cue probe, 1 invalid between and 1 invalid outside).
